# Pooled Segregant Sequencing Reveals Genetic Determinants of Yeast Pseudohyphal Growth

**DOI:** 10.1371/journal.pgen.1004570

**Published:** 2014-08-21

**Authors:** Qingxuan Song, Cole Johnson, Thomas E. Wilson, Anuj Kumar

**Affiliations:** 1 Department of Molecular, Cellular, and Developmental Biology, University of Michigan, Ann Arbor, Michigan, United States of America; 2 Departments of Pathology and Human Genetics, University of Michigan Medical School, Ann Arbor, Michigan, United States of America; Stanford University School of Medicine, United States of America

## Abstract

The pseudohyphal growth response is a dramatic morphological transition and presumed foraging mechanism wherein yeast cells form invasive and surface-spread multicellular filaments. Pseudohyphal growth has been studied extensively as a model of conserved signaling pathways controlling stress responses, cell morphogenesis, and fungal virulence in pathogenic fungi. The genetic contribution to pseudohyphal growth is extensive, with at least 500 genes required for filamentation; as such, pseudohyphal growth is a complex trait, and linkage analysis is a classical means to dissect the genetic basis of a complex phenotype. Here, we implemented linkage analysis by crossing each of two filamentous strains of *Saccharomyces cerevisiae* (Σ1278b and SK1) with an S288C-derived non-filamentous strain. We then assayed meiotic progeny for filamentation and mapped allelic linkage in pooled segregants by whole-genome sequencing. This analysis identified linkage in a cohort of genes, including the negative regulator *SFL1*, which we find contains a premature stop codon in the invasive SK1 background. The S288C allele of the polarity gene *PEA2*, encoding Leu409 rather than Met, is linked with non-invasion. In Σ1278b, the *pea2*-M409L mutation results in decreased invasive filamentation and elongation, diminished activity of a Kss1p MAPK pathway reporter, decreased unipolar budding, and diminished binding of the polarisome protein Spa2p. Variation between SK1 and S288C in the mitochondrial inner membrane protein Mdm32p at residues 182 and 262 impacts invasive growth and mitochondrial network structure. Collectively, this work identifies new determinants of pseudohyphal growth, while highlighting the coevolution of protein complexes and organelle structures within a given genome in specifying complex phenotypes.

## Author Summary

Cellular processes in eukaryotes are brought about through the contributions of large gene sets, and a continuing obstacle in studying these processes lies in the identification of critical constituent genes. The yeast pseudohyphal growth transition is an important example of a complex cellular growth transition. During pseudohyphal growth, yeast cells form connected chains or filaments, constituting a means of foraging for nutrients under conditions of nitrogen and/or glucose limitation. Yeast pseudohyphal growth has been studied for over two decades as a model of signaling systems controlling stress responses, cell shape, and fungal virulence. Hundreds of genes are required for pseudohyphal growth, however, and the critical genes that determine the filamentous phenotype have not been elucidated. Towards this goal, we implemented a genetic approach to identify alleles linked with the pseudohyphal growth phenotype. These studies identified previously unstudied variation in proteins functioning in a complex that controls cell polarity and in a protein of the mitochondrial inner membrane. This work indicates that proteins in complexes and organelles have coevolved within a given genome to yield distinct outputs and phenotype, while highlighting the application of an approach that is useful for the analysis of complex phenotypes in many eukaryotes.

## Introduction

The budding yeast *Saccharomyces cerevisiae* undergoes a pronounced growth transition in response to nitrogen limitation or glucose limitation, forming multicellular pseudohyphal filaments that can spread outward from a colony and/or invade the surface of a solid growth substrate [1], [2]. Yeast pseudohyphal filament formation is a presumed foraging mechanism, accomplished through underlying changes in cell adhesion, cell cycle progression, and budding [1], [3], [4]. During pseudohyphal growth, yeast cells remain physically connected after cytokinesis via mechanisms encompassing the regulated expression and shedding of the flocculin Flo11p [5]–[7]. Cells undergoing pseudohyphal growth exhibit increased apical growth through reorganization of the actin cytoskeleton, regulation of polarity proteins, and delayed G2/M progression [8]–[12].

The molecular basis of yeast pseudohyphal growth has been studied extensively as a model of conserved signaling pathways controlling cell morphogenesis and polarity. Furthermore, related processes of filamentous development in the principal opportunistic human fungal pathogen *Candida albicans* are required for virulence, and signaling pathways between the related yeasts are conserved [13]. Classic studies of pseudohyphal growth in *S. cerevisiae* have resulted most prominently in the identification of core pseudohyphal growth signaling modules encompassing the Kss1p mitogen-activated protein kinase (MAPK) cascade, the cAMP-dependent protein kinase A (PKA) pathway, and the AMP-activated protein kinase ortholog Snf1p [14]–[20]. The pseudohyphal growth MAPK cascade encompasses Ste11p, Ste7p, and the MAPK Kss1p [10], [14]. Kss1p phosphorylates the Ste12p transcription factor, resulting in dissociation of the negative regulatory Dig1p and Dig2p interactors and binding of a Ste12p-Tec1p heterodimer to target promoters, such as the *FLO11* promoter [21]–[23]. Tpk2p, a catalytic subunit of PKA, phosphorylates the Flo8p transcription factor, promoting Flo8p binding and transcriptional activation at the *FLO11* promoter and other regulatory sites [17], [24]–[26]. In response to glucose limitation, *FLO11* transcription is regulated by Snf1p; the Snf1p-Gal83p isoform promotes cell adhesion during invasive filamentation by antagonizing Nrg1p- and Nrg2p-mediated repression of *FLO11* [19], [27].

While the central components of these signaling pathways have been identified, the scope of the yeast pseudohyphal stress response is broad [28]–[33], and the mechanisms enabling these genes and gene products to drive pseudohyphal filamentation are incompletely defined, as are the genetic determinants within this gene set that underlie filamentation. To further dissect pseudohyphal growth pathways, we implemented a linkage study, coupling whole genome sequencing with pooled segregant analysis. The results present previously unidentified genetic determinants of yeast invasive growth and indicate the coevolution of proteins within complexes in driving phenotype.

## Results

### Pooled Segregant Analysis of Yeast Invasive Filamentation with Deep Sequencing

**Figure 1:**
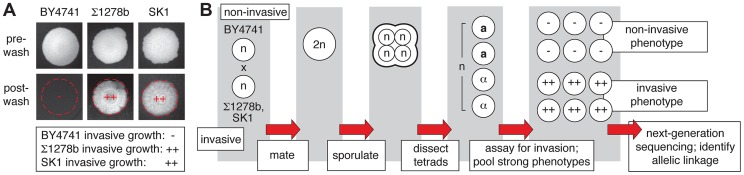
Overview of pooled segregant analysis to identify linkage with invasive growth phenotypes. A) Invasive growth phenotypes of the non-filamentous S288C-derived BY4741 strain and the filamentous Σ1278b and SK1 strains. Spotted cultures of the strains are shown on YPD medium before and after washing the surface of the agar plate under a gentle stream of water. Residual cells after washing are indicative of invasive growth. The degree of invasive growth is shown overlaid on each respective image and in the boxed inset, with “++” indicating strong invasive growth, and “−” indicating an absence of invasive growth. B) Schematic depicting the pooled segregant whole-genome sequencing approach.

For linkage analysis, we selected as parents the non-filamentous S288C-derived strain BY4741 and the filamentation-competent strains Σ1278b and SK1 [34], [35]. Filamentous-form growth in haploid strains is classically assessed using the plate-washing assay of Gimeno *et al.* [1] to identify pseudohyphal cells that have invaded the agar substrate. The invasive phenotype of each parent strain in this assay is indicated in Fig. 1A. The experimental design of the linkage study is presented in Fig. 1B. The non-invasive S288C-derived strain was mated with each of the filamentous Σ1278b and SK1 strains, and the resulting diploid strain from each cross was sporulated. Meiotic progeny from dissected tetrads were assayed for agar invasion by plate-washing, and spores indicating strongly non-invasive or invasive phenotypes were pooled for subsequent linkage analysis. Only spores resulting from complete meiosis were included in these phenotypic pools, and intermediate filamentation phenotypes were excluded from subsequent analysis to provide the greatest likelihood of identifying allelic variation with a strong effect on filament formation. Genomic DNA was extracted from each segregant pool and subjected to high-throughput sequencing that yielded greater than 100-fold coverage per pool.

### Identification of Allelic Variants Linked with Invasive Phenotype between a Non-filamentous S288C Derivative and Σ1278b

**Figure 2:**
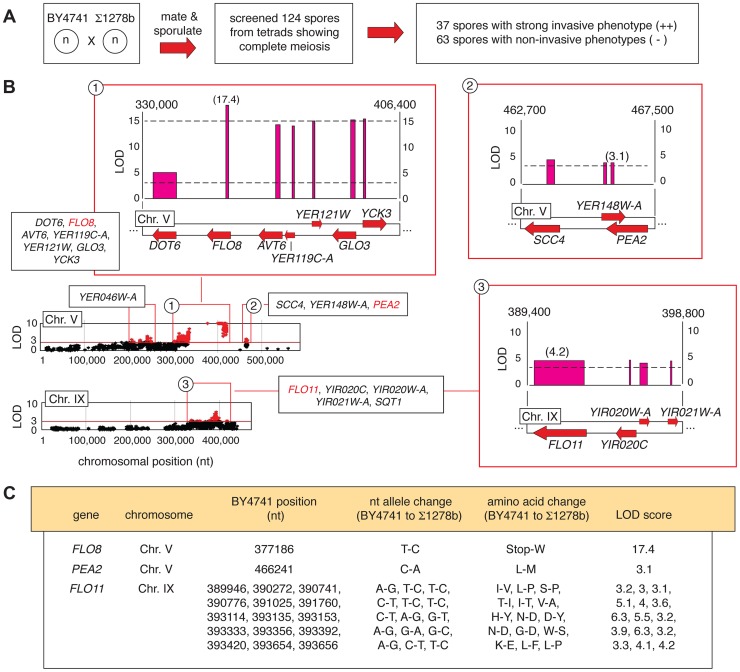
Identification of alleles linked with invasive growth in meiotic progeny from the BY4741-by-Σ1278b cross. A) Summary of phenotypic screening for invasive growth in haploid spores. B) Plots of non-synonymous allelic changes graphed by chromosomal location are presented, with allelic variation exhibiting a LOD score of greater than 3 highlighted in red. Clusters of alleles with LOD scores greater than 3 are boxed, and expanded blow-ups of loci on Chromosome V and IX housing the invasive growth determinants *FLO8*, *PEA2*, and *FLO11* are shown. Red arrows represent genes, with forward directionality indicating the Watson strand of the respective chromosome. LOD scores are plotted above each gene. The width of the LOD score bar for each gene corresponds to the chromosomal boundaries of the allelic changes observed in the respective gene coding sequence; a single nucleotide change between the sequences is indicated by a thin line. Chromosomal nucleotides delineating the boundaries of the highlighted region are shown at the upper right and left of each expanded plot. C) Specific nucleotide and amino acid changes between alleles of the *FLO8*, *PEA2*, and *FLO11* genes are indicated, along with the respective LOD score for each gene.

From the BY4741-by-Σ1278b cross, 31 complete tetrads (124 spores) were screened for agar invasion, identifying 37 strongly invasive spores and 63 non-invasive spores (Fig. 2A). The segregant pools were sequenced, and candidate determinants of the invasive phenotype were identified using a linkage LOD score of greater than 3 as an arbitrarily defined cut-off. Table S1 provides a listing of these alleles, encompassing only variants that are in protein-coding sequence and that are non-synonymous with respect to the encoded amino acid sequence. This allele set affects 50 genes in eleven linkage blocks physically located on seven yeast chromosomes. Figure S1 summarizes the available functional information for this gene set.

Representative plots of non-synonymous allelic variation with respective LOD scores are graphed in Fig. 2B for chromosomes V and IX, highlighting the pseudohyphal growth transcription factor gene *FLO8* and the flocculin effector gene *FLO11*. *FLO8* is a pseudogene in S288C-derived strains [36], and in this analysis, the BY4741 allelic variant containing a premature translational stop at codon 142 of the *FLO8* sequence yielded a LOD score greater than 17 (Fig. 2B and C). The *FLO11* locus exhibits fifteen allelic changes linked with invasive growth phenotypes (Fig. 2B and C). Previous studies identified allelic variation in *FLO11* sequence encoding amino- and carboxy-terminal regions linked with the ability to form biofilms on the surface of wine [37]. We recovered these as well as additional sites of DNA sequence variation in *FLO11*, with the Σ1278b-encoded alleles indicating linkage with strong invasive growth. The *FLO11* sequence contains an internal repeat region that is a source of allelic variation between some strains and colonies [38], [39]; however, we did not observe a change in the number of these repeats between BY4741 and Σ1278b. Collectively, the identification of these known pseudohyphal growth genes demonstrates the relevance of results obtained from our pooled segregant analysis.

### *PEA2* Allelic Variation Impacts Cell Morphology and MAPK Pathway Activity

To further identify important determinants of invasion, we screened candidates from Table S1 as follows: 1) we generated gene deletions and assayed for invasive growth phenotypes (Table S2), and 2) for genes yielding deletion phenotypes, we generated mutants with swapped alleles to identify genetic variants required for invasive growth in Σ1278b. In particular, we focused on alleles of genes that contributed to cell polarity, cell cycle progression, cell morphology, and cell responses to nitrogen/carbon limitation, as these are hallmark characteristics of filamentation.

**Figure 3:**
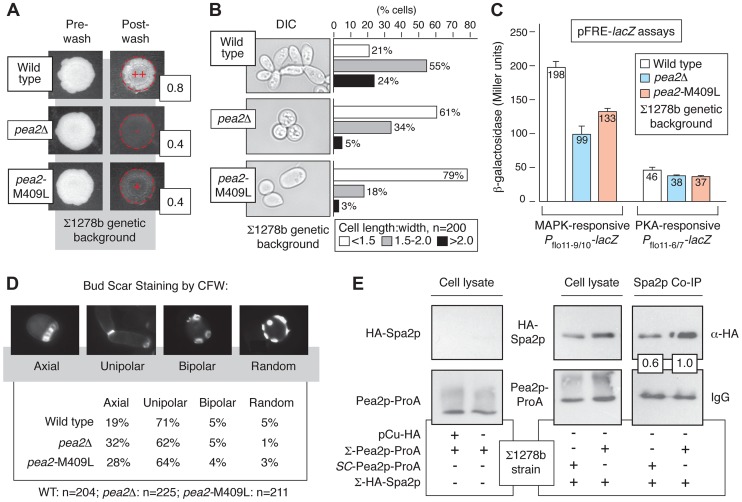
Variation at Pea2p residue 409 impacts invasive growth, budding, and polarisome interactions. A) Invasive growth of the wild-type Σ1278b strain, *pea2*Δ strain, and a strain containing the integrated *pea2*-M409L point mutation in the Σ1278b background was assayed by plate washing (++, strong invasive growth; +, invasive growth; −, lack of invasive growth). Invasive growth levels were quantified by determining the ratio of pixel intensity of images of the spotted cultures before and after plate washing; these ratios are indicated to the lower right of each spotted culture image post-washing. B) Cell morphology was assessed by determining the length-to-width ratio of 200 cells each for the indicated strains. Prior to imaging, cells grown on YPD medium were scraped into a suspension in liquid media. For length-width calculations, the longest cell axis was selected for cell length measurements. C) Activity of MAPK and PKA signaling pathways in *pea2* mutants. The bar graphs indicate activity of plasmid-based cAMP-dependent PKA and pseudohyphal growth MAPK pathway-responsive *P*_flo11_-*lacZ* reporters. The PKA pathway-responsive *P*_flo11_ reporter contains a fragment of the *FLO11* promoter (*P*_flo11-6/7_) 21.0–21.4 kb from the *FLO11* start codon, and the Kss1p MAPK-responsive *P*_flo11_ reporter contains a promoter fragment (*P*_flo11-9/10_) 21.6–22.0 kb upstream of the *FLO11* start codon. β-galactosidase activity was measured from cells growing exponentially in SC-Ura medium. Error bars indicate standard deviations of three independent measurements. D) Budding patterns were analyzed for the indicated strains by calcofluor white staining of invasive cells recovered from the agar. Sample images of the observed budding patterns are presented. Budding patterns of the *pea2*Δ and *pea2*-M409L mutants are distinct from wild type, with *p*-values of <0.01 and <0.001, respectively, by Pearson's chi-squared test. E) Western blots indicate co-immunoprecipitation of Spa2p by Pea2p variants in the Σ1278b background. For this analysis, chromosomal *PEA2* and *pea2*-M409L were tagged at the carboxy terminus by integration of a cassette encoding ProA. Spa2p tagged at its amino terminus with the hemagglutinin (HA) epitope was expressed from a plasmid using a copper-inducible promoter. The binding affinity between Pea2p and Spa2p was revealed by IgG pull-down using Pea2p-ProA as bait. Pea2p and Spa2p levels were normalized against levels observed in total cell lysate. The ratio of recovered Spa2p by the S288C-encoded Pea2p-L409 variant is presented relative to the ratio of Spa2p recovered by Σ1278b-encoded Pea2p-M409; thus, the boxed ratios indicate that Pea2p-L409 recovered 60% the amount of Spa2p co-immunoprecipitated with Pea2p-M409.

By this approach, we identified variation in *PEA2* as an important part of the genetics underpinning invasive growth. Pea2p localizes to sites of polarized growth as a component of a protein complex, termed the polarisome [40],[41]. *PEA2* is required for wild-type invasive growth, mating projection formation, and bipolar bud site selection in diploids [42], [43]. In the filamentous Σ1278b strain, *PEA2* codon 409 specifies methionine rather than the leucine residue encoded in the S288C-derived reference genome. The *pea2*-M409 allele was linked with invasive growth, and generation of an integrated site-specific mutation (*pea2*-M409L) reconstituting the S288C-encoded *PEA2* allele in Σ1278b resulted in decreased invasive growth (Fig. 3A). Relative to wild type Σ1278b, the cell morphology of the *pea2*-M409L mutant is altered, exhibiting decreased elongation (Fig. 3B); over a population of 200 cells, the percentage of *pea2*-M409L cells with a length:width ratio of less than 1.5 was nearly four-fold the corresponding percentage in a wild type strain. In addition, the *pea2*-M409L mutant is impaired in Kss1p MAPK signaling activity. The Kss1p kinase activates the Ste12p/Tec1p transcription factor complex, which recognizes a regulatory element (FRE) in the *FLO11* promoter. The plasmid-based *P*_flo11-9/10_-*lacZ* construct contains the Ste12p/Tec1p-responsive region of the *FLO11* promoter fused to *lacZ* [6], and, by this reporter, the *pea2*-M409L mutant yields significantly decreased Ste12p/Tec1p-dependent transcriptional activation of *FLO11* relative to wild-type Σ1278b (Fig. 3C). In contrast, the *pea2*-M409L mutation results in wild-type levels of a similarly designed *FLO11* promoter fusion responsive to the PKA pathway effector Flo8p (Fig. 3C) [6].

### Genetic Variation in *PEA2* Modulates Bud Site Selection and Spa2p Binding

Under conditions of vegetative growth haploid yeast cells bud in an axial pattern, with new buds emerging adjacent to the preceding bud site [44]. Haploid cells undergoing pseudohyphal growth, however, adopt a predominantly unipolar budding pattern wherein the first bud forms distal to the original cell division site, and subsequent buds cluster in the distal pole [1], [10]. Here, we find that in the Σ1278b background the *pea2*-M409L mutant, corresponding to the S288C-encoded *PEA2* allele, exhibited a decrease in unipolar budding and an increase in axial budding relative to wild type (*p*<0.001), with levels intermediate between an otherwise isogenic wild-type strain and a *pea2*Δ mutant (Fig. 3D). For this analysis, we examined a population of invasive cells exhibiting three or more bud scars, such that patterns of axial, unipolar, bipolar, and random budding could be reliably distinguished [44], [45]. This budding phenotype was evident in invasive cells, but not in an equally sized population of cells scraped from the surface of an agar plate. Previous studies have indicated that the majority of bud sites are distal in a *pea2*Δ mutant [12]; results here also indicate that the majority of bud sites are distal in *pea2* mutants, but budding pattern analysis does indicate that Pea2p residue 409 impacts unipolar budding in invasive haploid cells.

In the polarisome complex, Pea2p binds the scaffolding protein Spa2p, a large coiled-coil domain-containing protein required for polarisome function [41], [46]. Here, we assessed the possibility that allelic variation at the *PEA2* locus impacts Spa2p binding, using Protein A (ProA)-tagged Pea2p variants to recover by co-immunoprecipitation Spa2p tagged at its amino terminus with the hemagglutinin (HA) epitope. In the Σ1278b strain, the Pea2p-M409-ProA variant recovered more HA-Spa2p than the Pea2p-L409-ProA variant, representing the S288C-encoded *PEA2* allele (Fig. 3E).

### Analysis of Genetic Variation Linked with Invasion between BY4741 and SK1

**Figure 4:**
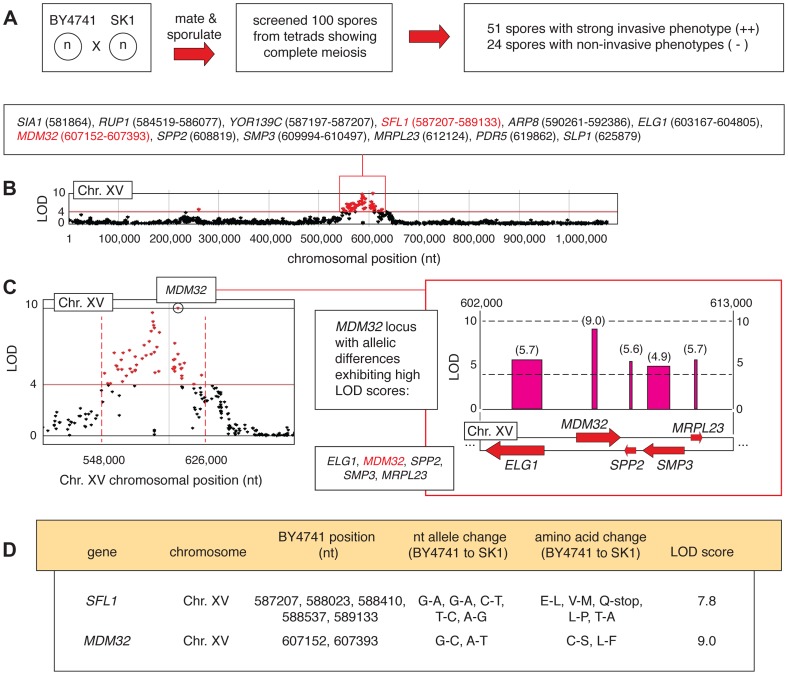
Identification of allelic linkage with invasive growth from the BY4741-by-SK1 cross. A) Summary of phenotypic screening results. B) A chromosomal plot indicating non-synonymous allelic variation and LOD scores is presented for chromosome XV, highlighting a region of clustered high LOD scores (>4) around the *SFL1* and *MDM32* loci. This region is boxed in red, and the constituent genes are indicated with the nucleotide positions of the allelic difference; for genes that contain multiple allelic differences, a nucleotide range is listed encompassing the respective residues. C) The *MDM32* locus is highlighted in a plot of LOD score by chromosomal position, and the inset box indicates the orientation of *MDM32* and neighboring genes on chromosome XV. LOD scores are indicated for each gene in the boxed region, and as in Figure 2, the width of the LOD score bar indicates the chromosomal boundaries of the allelic changes. D) Specific nucleotide and amino acid changes between BY4741- and SK1-encoded alleles of the *SFL1* and *MDM32* genes are indicated, along with the respective LOD score for each gene.

The BY4741-by-SK1 cross was implemented as described in Experimental Procedures, and phenotypic analysis of meiotic progeny identified 51 and 24 strongly invasive and non-invasive spores, respectively (Fig. 4A). Subsequent deep-sequencing of the phenotypic pools identified allelic variation linked with invasive growth phenotypes within eleven separated locus blocks encompassing 88 genes exhibiting non-synonymous changes and a LOD score of greater than 4 (Table S3). A functional breakdown of these genes is indicated in Figure S2. In this analysis, we used a higher LOD score relative to the Σ1278b-by-BY4741 cross in order to limit the number of selected allelic variants to a manageable size for further study, as the SK1 and BY4741 genomes are more divergent (99.5% sequence identity) than the Σ1278b and BY4741 genomes (99.7% identity) [47]. Very few allelic variants linked with invasive growth in Σ1278b were also identified in SK1, aside from a few sequences near *FLO8* that are unlikely to be causative. The set of identified alleles was primarily distinct between the two linkage studies, and deletion phenotypes for tested genes in SK1 are indicated in Table S4.

In haploid spores from this cross, genetic variation is most strongly linked with the invasive growth phenotype over a region of roughly 80,000 bp on chromosome XV, encompassing *SFL1* (Fig. 4B and C). *SFL1* encodes a transcriptional repressor of pseudohyphal growth that functions by binding to the *FLO11* promoter, thereby blocking transcriptional activation [24], [25], [48]–[51]. Consistent with its function in repressing *FLO* gene expression, deletion of *SFL1* results in exaggerated invasive growth [28], [32], [48]. Interestingly, the SK1 strain contains an allelic variant of *SFL1* with respect to S288C-derived strains, resulting in the conversion of codon 477 (CAA encoding glutamine) to a TAA stop codon (Fig. 4D). This premature stop codon truncates *SFL1* prior to the sequence encoding a domain (AA 571–658) that is strongly similar to a conserved region in Myc oncoproteins [48]. Previous studies have identified hyperactive filamentation in a mutant of the CEN.PK 113-7D background upon introduction of a premature translational stop at *SFL1* codon 320 (Q320-stop) [51]. Here, we found that allelic variation in *SFL1*, encompassing a premature stop codon (C1430T, Q477-stop) in the SK1 background, is linked to the aggressively invasive phenotype of SK1 relative to BY4741.

### Allelic Variation in *MDM32* Affects Mitochondrial Morphology and Invasive Growth

In the BY4741-by-SK1 cross, allelic variation in *MDM32* was linked to invasive growth more strongly than any other identified locus, with a LOD score of 9. As indicated in Fig. 4B–D, *MDM32* is found on chromosome XV, and relative to BY4741, the SK1 allele of *MDM32* encodes Ser182 and Phe262 rather than Cys and Leu, respectively. *MDM32* encodes a protein complex subunit of the mitochondrial inner membrane required for membrane organization, the maintenance of elongated mitochondrial morphology, and mitochondrial DNA nucleoid stabilization [52]. Mitochondrial function is required for pseudohyphal growth, as filamentation-competent strains of *S. cerevisiae* containing a deleted version of the mitochondrial genome are unable to form pseudohyphae [53], [54]; however, a role for Mdm32p in enabling invasive growth remains to be identified.

**Figure 5:**
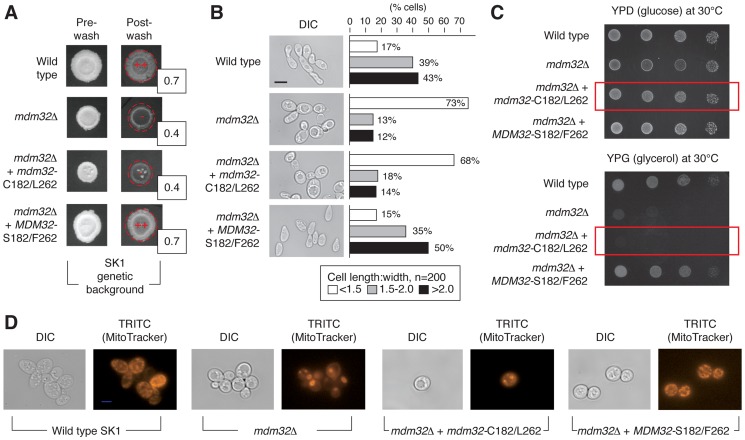
Phenotypic consequences of variation in Mdm32p residues 182 and 262. A) Invasive growth assays of wild-type and *mdm32* mutants in the SK1 background were performed as described previously. Invasive growth levels were quantified by determining the ratio of the pixel intensity of images of the spotted cultures before and after plate washing, and these ratios are indicated in the boxes to the lower right of the spotted cultures post-washing. B) Introduction of the BY4741-encoded *MDM32-C182/L262* allele into SK1 results in decreased cell elongation relative to wild type. In this analysis, cells were scraped from colonies into a suspension for imaging of cell morphology as described previously. C) A strain of SK1 carrying the S288C-encoded allele of *MDM32* exhibits a growth defect on medium containing the non-fermentable carbon source glycerol. Cells from the indicated strains were grown overnight in glucose- containing medium, and subsequently, 10-fold serial dilutions were spotted onto plates containing glucose (YPD) or glycerol (YPG) as a carbon source. YPD plates were incubated for 2 days and YPG plates for 3 days at the indicated temperatures. D) Analysis of mitochondrial network structure in a mutant strain of the SK1 background carrying the BY4741-encoded *MDM32-C182/L262* allele. As visualized by staining with a mitochondrial dye, the mitochondrial network is compact and collapsed in a strain deleted for *MDM32* and in the SK1 mutant carrying the BY4741 allele.

To determine the effect of *MDM32* allelic variation on pseudohyphal growth, we replaced the SK1-encoded *MDM32*-C546/T787 allele, specifying Mdm32p-S182/F262, with BY4741-encoded *MDM32*-G546/A787, specifying Mdm32p-C182/L262, in the SK1 genetic background. This allelic swap decreased invasive growth in SK1, and agar invasion was rescued upon reintroduction of the native SK1-encoded *MDM32* allele (Fig. 5A). The SK1 mutant containing the BY4741-encoded allele of *MDM32* exhibited a more rounded cell morphology, with the percentage of cells displaying a cell length:width ratio of less than 1.5 increasing from 17% in wild-type SK1 cells to 68% in the SK1 mutant (Fig. 5B). Reintroduction of SK1-encoded *MDM32* recovered levels of cell elongation similar to wild type.

To assess the impact of this allelic variation on mitochondrial function, we grew the SK1 mutant with the BY4741 allele of *MDM32* on medium containing non-fermentable glycerol as the sole carbon source. As shown in Fig. 5C, the allele-swapped SK1 mutant grows poorly in glycerol-containing media, indicating that oxidative phosphorylation is impaired. The structure of the mitochondrial network is also perturbed upon introduction of the BY4741 allele of *MDM32* in the SK1 background. Using the mitochondrion-specific MitoTracker fluorescent dye, which diffuses passively across the plasma membrane and concentrates in active mitochondria by membrane potential, we can visualize a compact and collapsed mitochondrial network in SK1 cells containing the BY4741-encoded Mdm32p-C182/L262 variant, similar to that observed in *mdm32*Δ (Fig. 5D). *MDM32* is a paralog of *MDM31*, and the encoded proteins have been found to interact, albeit transiently and weakly, as components of protein complexes at the mitochondrial inner membrane [52]. We, therefore, assessed the effect of allelic variation at *MDM32* on Mdm31p binding; however, we observed no difference in the recovery of Mdm31p by co-immunoprecipitation between the respective Mdm32p variants (Figure S3). In sum, *MDM32* is a determinant of invasive growth, and replacement of the native SK1 allele of *MDM32* with the BY4741-encoded allele yields a mutant filamentous growth phenotype.

## Discussion

**Figure 6:**
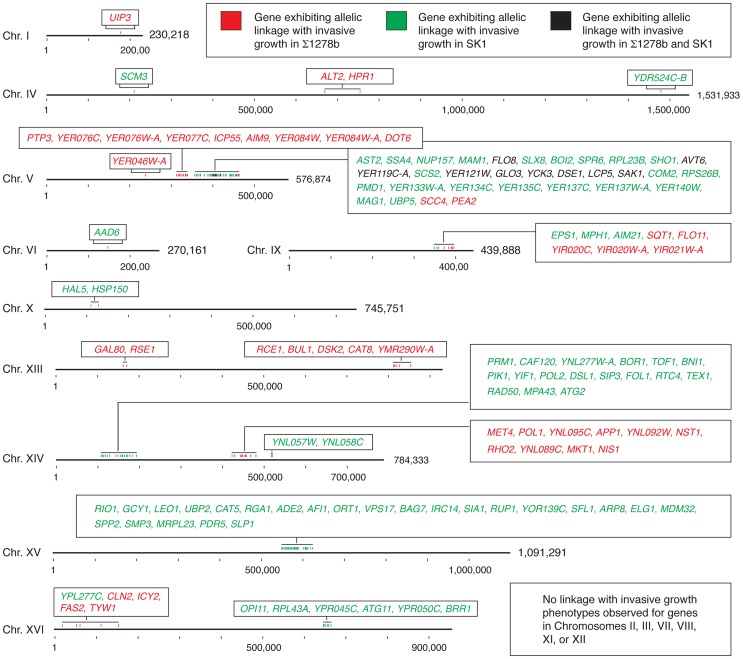
Chromosomal location of genes exhibiting allelic linkage with invasive growth from the BY4741 cross with Σ1278b and SK1, respectively. Genes within a chromosomal region are listed in the boxes with color-coding to indicate the respective cross from which the linkage was identified. Hash marks indicate 100,000 bases, and the full length of each chromosome is indicated.

The linkage analysis presented here identifies a broad gene set contributing to pseudohyphal growth (Fig. 6). The pattern of allelic linkage indicates a large number of determinant loci underlying invasive growth, consistent with results from systematic single-gene deletion and overexpression studies [28], [31], [32]. Within this gene set, components of the polarisome and mitochondria play important roles in enabling invasive growth. We report here that Pea2p residue 409 impacts bud site selection in haploid cells undergoing invasive growth, although the effect is less pronounced in determining initial distal-versus-proximal budding in virgin mother cells. Pea2p residue 409 lies distinct from the Pea2p coiled-coil region between residues 236 and 327 and is important for Spa2p binding. Spa2p interacts with Ste11p and Ste7p from the Kss1p MAPK pathway, providing a mechanism for polarisome-mediated regulation of Kss1p MAPK activity [41]. Our results further indicate that nuclear-encoded Mdm32p is required for invasive growth in SK1, and that residues 182 and 262, located outside of the mitochondrial pre-sequence (AA 1–102) and at the boundary or outside of a transmembrane domain (AA 161–184 and 636–653), are important in enabling invasion, as well as in determining aerobic respiratory function and mitochondrial morphology. Mdm32p is proposed to function cooperatively with other inner membrane proteins and components of the outer mitochondrial membrane in the maintenance of mitochondrial morphology [52], potentially through cytoskeletal interactions that may be affected by variation at these sites.

This analysis highlights two additional points. First, the non-filamentous BY4741 background is not uniformly repressive with respect to pseudohyphal growth. From the Σ1278b and SK1 crosses, we identified three and five blocks of allelic variation, respectively, in BY4741 linked with the invasive growth phenotype; full listings of the encompassed alleles with respect to each cross are presented in Tables S5 and S6. Alleles in S288C-derived strains that promote pseudohyphal growth are antagonized by alleles that repress filament formation, such as the pseudogene form of *FLO8*; similarly, alleles in SK1 linked with the non-invasive phenotype may be offset by alleles that promote invasion, such as the *SFL1* allele containing a premature stop codon. Second, the identified allelic variation in *PEA2* and *MDM32* and the allele-swapping experiments performed here indicate that within a given genome, functionally interacting genes coevolve to impact phenotype. The majority of genetic variation linked with invasive phenotype in this study involves site-specific changes that do not create pseudogenes. Alleles of these genes yield functional proteins within the respective genomic contexts; however, a given allele results in a hypomorphic phenotype when introduced into a non-native strain. It should be noted that the BY4741-encoded allele of *PEA2* may be viewed as being pseudohyphal competent, as Liu *et al.* [36] reported that the introduction of Σ1278b-encoded *FLO8* in a S288C-derived strain is sufficient to enable at least some degree of pseudohyphal growth. The data here suggest that partner genes have likely co-evolved with genes such as *PEA2* and *MDM32*, and the resulting protein complexes are, thus, an important determinant of cell phenotype. These findings highlight the utility in studying these complexes as a whole, in supplement to individual proteins, in order to accurately identify the functions and properties that specify phenotype.

It is interesting that the studies here indicated very little overlap between alleles linked with invasive growth in the Σ1278b and SK1 strains with respect to BY4741 (Fig. 6). Studies mapping quantitative trait loci (QTL) in a cross of the laboratory strain BY4716 and the vineyard strain RM-11 identified hotspots impacting gene expression, protein abundance, and small molecule-dependence [55]–[58]. These hotspots were principally due to alleles in the BY4716 background, leading Ronald and Akey [59] to suggest that the causative polymorphisms may occur at low frequency. The non-overlapping allele sets identified in our analysis are not suggestive of hotspots, but rather highlights the substantial importance of epistatic interactions in determining the sum filamentous phenotype resulting from variant alleles in the haploid segregants. These epistatic interactions likely represent instances of gene coevolution, which has been suggested to occur at an elevated rate for genes encoding proteins of shared biological functions and/or for proteins that have coevolved between species [60], [61]. Clark *et al.* [60] have analyzed the rate of covariation for pairs of proteins over evolutionary time, and by this analysis, polarisome components as a whole do not exhibit statistically significant evidence of covariation, although many mitochondrial complexes do yield signature indicating evolutionary rate covariation. Further analyses of individual protein pairs from the strains used in our study will be necessary to identify a set of coevolved proteins that drive the filamentous growth phenotype.

In summary, we used pooled segregant whole-genome sequencing to dissect gene networks that determine yeast pseudohyphal growth. This analysis identified allelic variation in the known pseudohyphal growth genes *FLO8* and *FLO11*, while also revealing variation in the negative regulator *SFL1*, the coding sequence of which contains a premature stop codon in the invasive SK1 background. We further found that amino acid 409 in the polarisome protein Pea2p is a site of allelic variation critical for the protein's ability to signal through the Kss1p MAPK pathway, establish unipolar budding during pseudohyphal growth, and bind the Spa2p polarisome scaffold. Linkage analysis identifies variation in *MDM32* as a determinant of invasive growth between S288C derivatives and the SK1 strain; the 182 and 262 residues are sites of variation and contribute to Mdm32 function in aerobic respiration and invasive growth.

## Materials and Methods

### Yeast Strains and Growth Conditions

A listing of yeast strains and plasmids used in this study is provided in Tables S7 and S8. Haploid deletion mutants were constructed by PCR-mediated gene disruption using pFA6a-KanMX6 or pUG72 [62], [63]. Yeast strains were propagated on rich YPD medium (1% yeast extract, 2% polypeptone and 2% glucose) medium or synthetic medium as described [64]. Yeast invasive growth was assayed on YPD medium.

### Pooled Segregant Analysis

The statistical modeling used to derive the probabilities of identifying linkage are described by Birkeland *et al.* [65]. Following mating as indicated in Fig. 1, resulting strains were sporulated and asci were dissected. The dissected spores were grown overnight at 30°C and were individually tested for mating type. Spores resulting from complete meiosis (four viable spores with two each of the α and **a** mating types) were then used for whole genome sequencing. Each spore was assigned to invasive or non-invasive pools based on its invasive growth phenotype. To ensure equal representation of all segregants in a pooled population, each haploid strain was grown overnight at 30°C in individual 4 ml YPD cultures. The OD_600_ of the cultures was determined and used to calculate the appropriate volume of each strain so that upon mixing, we would achieve equal numbers of cells. A mate-pair library with 300-bp fragments was prepared for each of the phenotypic pools, and each library sequenced as paired-end reads using the Illumina Genome Analyzer (University of Michigan DNA Sequencing Core). Sequence analysis was performed as described [65]. To obtain an estimate of the recombinant and non-recombinant spore counts in each phenotypic pool for a given observed sequence variant, we multiplied the number of spores in the pool by the fraction of sequence reads from that pool that matched the corresponding allele variant. These values were then used in standard LOD score calculations.

### Analysis of Yeast Budding Pattern

Budding patterns of invasive cells were determined as previously described [44], [45]. In brief, equal concentrations of mid-log phase cultures were spotted onto YPD plates and incubated for 7 days at 30°C; surface cells were subsequently washed off under a gentle stream of water. Residual invaded cells were recovered from the agar using a sterile toothpick. Cells were washed twice in sterile water and were stained with 2 µg/ml calcoflour white. Bud scars were visualized by fluorescence microscopy. Cells with more than three bud scars were examined. Budding patterns were determined by criteria previously described [45]. Budding patterns were divided into four sub-groups: axial, bipolar, unipolar and random. The axial pattern was defined as a long chain of bud scars on the proximal cell pole. Cells with a cluster of scars exclusively at the distal pole were classified as exhibiting unipolar budding. A pattern of medial bud scars was scored as random budding, whereas cells with bud scars equally distributed on both proximal and distal poles were classified as undergoing bipolar budding. For these analyses, 200–250 cells from each strain were scored.

#### Plasmid construction

For expression of Σ1278b alleles in null mutants, yeast open reading frames of the Y826 background with 1 kb of upstream sequence and 300 bp of downstream sequence were cloned into a Gateway vector by standard methods of recombination-based Gateway cloning (Invitrogen) [66], [67]. For fusing enhanced green fluorescent protein (eGFP) and tandem affinity purification (TAP) cassettes to the carboxyl-terminus of the budding yeast Mdm31p and Mdm32p proteins, Gateway plasmids 416-GPD-ccdB-EGFP and 414-GPD- ccdB-TAP were used [68]. Plasmid pCu-Spa2p-3xHA-*URA3* was modified from pCu-3xHA-*URA3* using standard restriction enzyme digestion and ligation-based techniques. The *Sal*I and *Hind*III restriction sites of this plasmid was used to integrate the *SPA2* open reading frame between the copper-inducible *CUP1* promoter and 3xHA/GFP tag.

#### Integrated point mutations

Allelic variants of *PEA2* were generated in the Σ1278b background as integrated point mutations. A *URA3* cassette was amplified from the pUG6 plasmid and then used to replace the *PEA2* open reading frame in Y826. Subsequently, 5-FOA-mediated counter selection [69] was applied to replace the integrated *URA3* cassette with a 500 bp cassette encompassing the *PEA2* allelic change (M409L), recreating the S288C-derived allele in the Σ1278b background. The integrated point mutation was confirmed by sequencing of an amplified PCR fragment using the University of Michigan Sequencing Core.

#### Yeast invasive growth assays

Invasive growth of haploid strains was determined by the standard plate washing assay of Gimeno *et al.* [1]. Mid-log phase cultures were spotted onto YPD plates and incubated for 7 days at 30°C, and surface cells were subsequently washed off under a gentle stream of water. Residual cells from the spotted culture on the plate were imaged using a Nikon Eclipse 80i upright fluorescence microscope.

#### FRE-*lacZ* assays

Plasmids pLG669-Z FLO11-6/7 and -9/10 were transformed into designated yeast strains by standard protocols [64]. Cells were grown in SC-Ura media overnight, and then inoculated into fresh media to an OD_600_ of approximately 0.2. Cultures were subsequently grown for 3–4 hours to an OD_600_ of approximately 0.8–1.0. β-galactosidase activity was determined with the use of ortho-nitrophenyl-β-galactoside as a substrate (Sigma Aldrich) [70], [71].

#### Assays for respiratory activity

Respiratory ability was assessed using YPG plates with glycerol as a non- fermentable carbon source. Cells were pre-grown overnight and then inoculated into fresh SC medium to an OD_600_ of approximately 0.2; culture were subsequently incubated an additional 3 hours to an OD_600_ of approximately 0.8. The same amount of cells were serially diluted and spotted onto YPD and YPG (1% yeast extract, 2% polypeptone and 2% glycerol) plates, and incubated for 2 days at 30°C.

#### Fluorescence microscopy and image processing

For live-cell imaging, spotted cultures were grown in YPD plates for 7 days at 30°C. After washing the surface of the plates with a gentle stream of water, cells that had invaded the agar were recovered using a steel scalpel, and the extracted cells were washed twice with distilled water. Images were taken using a Nikon Eclipse 80i upright fluorescence microscope. To determine budding patterns, cells were stained with Calcofluor White as previously described [45]. Over 250 cells were counted twice for each strain. Statistics were analyzed using the ANOVA approach. To reveal mitochondrial morphology, living cells were stained with 50 nM MitoTracker Red, and the staining pattern was visualized by fluorescence microscopy [72].

#### Immunoprecipitation studies

*PEA2* alleles were generated by tagging with ProA on the C-terminus in wild type and *pea2-M409L* mutants in the Σ1278b strain. The *SPA2* open reading frame was cloned into pCu-3HA-URA, such that *SPA2* was expressed from a copper-inducible promoter as a fusion protein with three copies of the hemagglutinin epitope at its amino terminus. The resulting plasmids pCu-3HA-ScSPA2 and pCu- 3HA-ΣSPA2 were introduced by transformation into both wild type and *pea2-M409L* mutants. The binding affinity between selected proteins was detected by co-immunoprecipitation. For native immunoprecipitation, 2 OD units of cells were lysed in 1 ml lysis buffer (50 mM Tris-HCl [pH 7.5], 150 mM NaCl, 2 mM EDTA, 0.5% Triton X-100, 1 mM PMSF, and Complete EDTA-free protease inhibitor [Roche]) with glass beads. After centrifugation at 13,000 g for 10 min, the resulting supernatant was incubated with protein G-Sepharose 4 Fast Flow (GE Life Tech) for 2 hr at 4°C. After washing the Sepharose with lysis buffer six times, the bound materials were eluted by boiling the Sepharose in SDS-PAGE loading buffer. The resulting eluate was analyzed by Western blotting with designated antibodies (anti-HA, anti-Protein A and anti-GFP). Blots were developed using the SuperSignal West Dura Extended Duration Substrate (Thermo Scientific).

## Supporting Information

Figure S1 Functional characterization of genes exhibiting allelic linkage with invasive growth in the BY4741 cross with Σ1278b. Genes from the analysis that have been previously identified as exhibiting filamentous growth phenotypes upon deletion [32] are listed to the left. The remaining genes are listed to the right, with genes of unknown functions and genes with functions that may be related to pseudohyphal growth indicated separately. Functions are drawn from data deposited in the Saccharomyces Genome Database as of publication. The chromosome in which the gene is located is indicated in parentheses. It should be noted that this list encompasses genes that may only exhibit linkage because of their close proximity to an important allelic determinant.
(TIF)

Figure S2 Functional characterization of genes exhibiting allelic linkage with invasive growth in the BY4741 cross with SK1. Genes from the analysis that were identified as exhibiting filamentous growth phenotypes upon deletion [32] are listed to the left. The remaining genes are listed to the right, with genes of unknown functions and genes with functions that may be related to pseudohyphal growth indicated separately. Functions are drawn from data deposited in the Saccharomyces Genome Database as of publication. The chromosome in which the gene is located is indicated in parentheses. It should be noted that this list encompasses genes that may only exhibit linkage because of their close proximity to an important allelic determinant.
(TIF)

Figure S3 Binding of Mdm31p by BY4741- and SK1-encoded Mdm32p variants. The *MDM32* open reading frames from SK1 and BY4741 were cloned into a GPD-eGFP Gateway plasmid, such that the *MDM32* sequence was expressed from the *GPD1* promoter as an in-frame 3′-fusion to sequence encoding enhanced GFP. *MDM31* was cloned into the GPD-TAP plasmid, yielding a fusion of the tandem affinity purification (TAP) tag to the carboxy terminus of Mdm31p upon expression from the *GPD1* promoter. The resulting plasmids were transformed into *mdm32*Δ mutants. The binding affinity between Mdm32p-GFP and Mdm31p-TAP was revealed by IgG pull-down using Mdm31p-TAP as bait. No significant difference in binding between Mdm31p-TAP and the respective Mdm32p-GFP variants was observed.
(TIF)

Table S1 Genes exhibiting allelic linkage (LOD>3) from the BY4741-by-Σ1278b cross. Alleles in a single chromosome are demarcated by a double line; groupings of alleles within a possible linkage block are separated by a single line.
(DOCX)

Table S2 Genes assayed for deletion phenotypes with respect to invasive growth in Σ1278b. Deletion phenotypes were assessed using standard plate-washing assays.
(DOCX)

Table S3 Genes exhibiting allelic linkage (LOD>4) from the BY4741-by-SK1 cross. Alleles grouped in a single chromosome are indicated by a double line; groupings of alleles within a single linkage block are indicated with a single line.
(DOCX)

Table S4 Genes assayed for deletion phenotypes with respect to invasive growth in SK1. Deletion phenotypes were assessed for invasive growth by plate-washing assays.
(DOCX)

Table S5 Alleles with variation between BY4741 and Σ1278b where the BY4741-encoded allele exhibits linkage with the invasive phenotype. Alleles within a given chromosome are separated by a double line.
(DOCX)

Table S6 Alleles with variation between BY4741 and SK1 where the BY4741-encoded allele exhibits linkage with the invasive phenotype. Alleles within a given chromosome are separated by a double line.
(DOCX)

Table S7 Strains used in this study.
(DOCX)

Table S8 Plasmids used in this study.
(DOCX)
