# Supplementary figures and images for: Pooled Segregant Sequencing Reveals Genetic Determinants of Yeast Pseudohyphal Growth
Source: PLoS Genet. 2014 Aug 21;10(8):e1004570. doi: 10.1371/journal.pgen.1004570 (PMC4140661; doi:10.1371/journal.pgen.1004570)

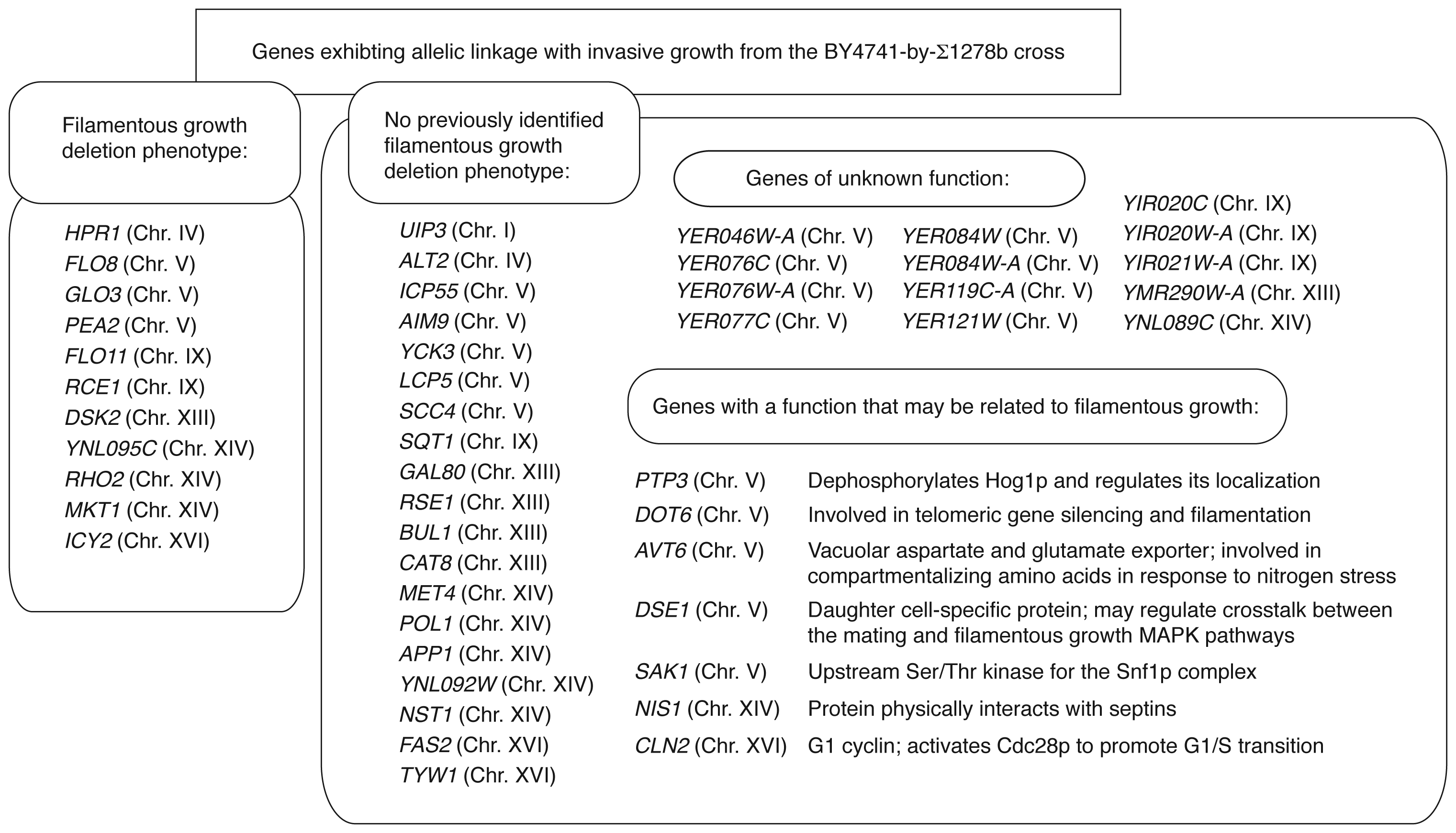

Supplement: Figure S1 — Functional characterization of genes exhibiting allelic linkage with invasive growth in the BY4741 cross with Σ1278b. Genes from the analysis that have been previously identified as exhibiting filamentous growth phenotypes upon deletion [32] are listed to the left. The remaining genes are listed to the right, with genes of unknown functions and genes with functions that may be related to pseudohyphal growth indicated separately. Functions are drawn from data deposited in the Saccharomyces Genome Database as of publication. The chromosome in which the gene is located is indicated in parentheses. It should be noted that this list encompasses genes that may only exhibit linkage because of their close proximity to an important allelic determinant. (TIF) [file pgen.1004570.s001.tif]

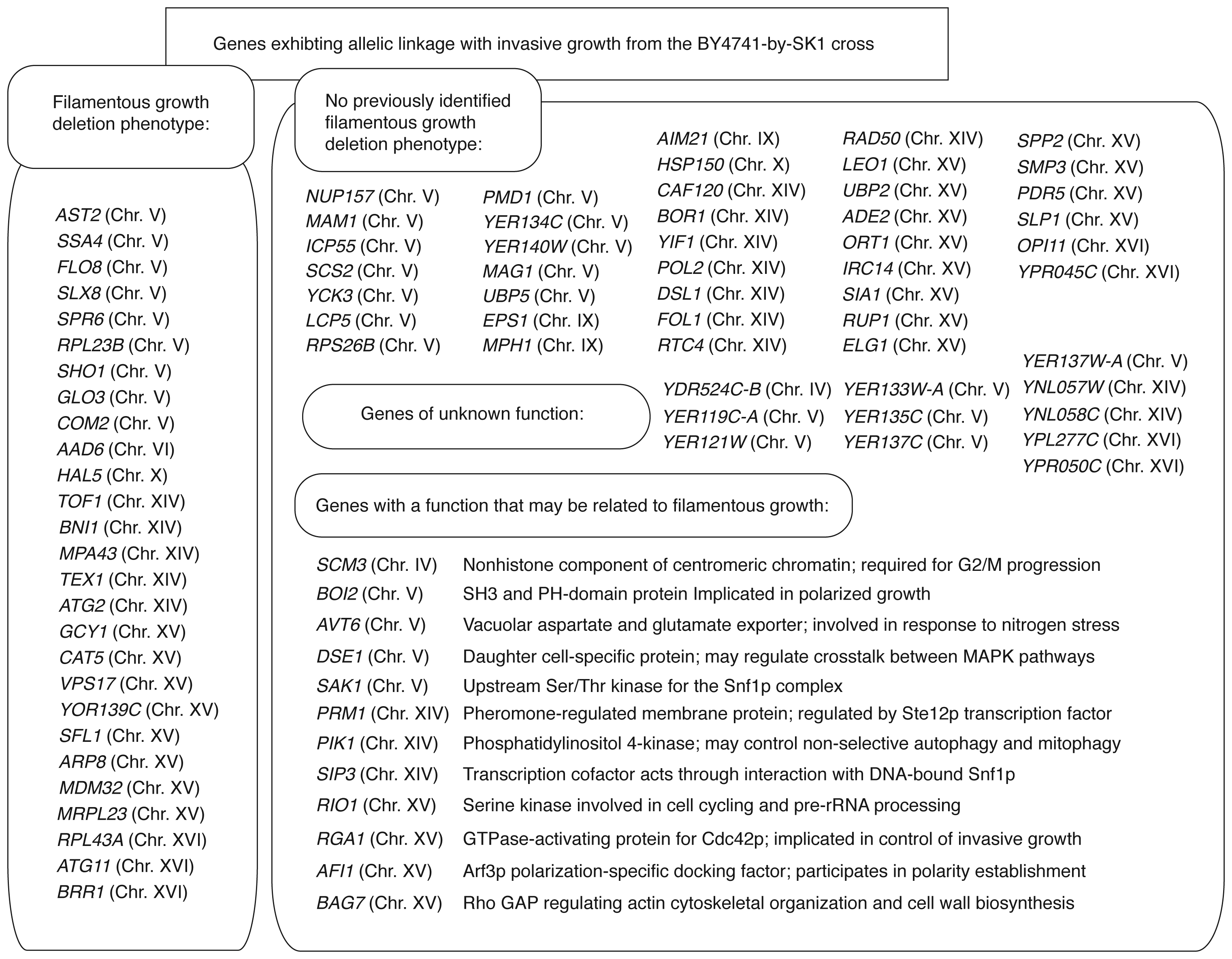

Supplement: Figure S2 — Functional characterization of genes exhibiting allelic linkage with invasive growth in the BY4741 cross with SK1. Genes from the analysis that were identified as exhibiting filamentous growth phenotypes upon deletion [32] are listed to the left. The remaining genes are listed to the right, with genes of unknown functions and genes with functions that may be related to pseudohyphal growth indicated separately. Functions are drawn from data deposited in the Saccharomyces Genome Database as of publication. The chromosome in which the gene is located is indicated in parentheses. It should be noted that this list encompasses genes that may only exhibit linkage because of their close proximity to an important allelic determinant. (TIF) [file pgen.1004570.s002.tif]

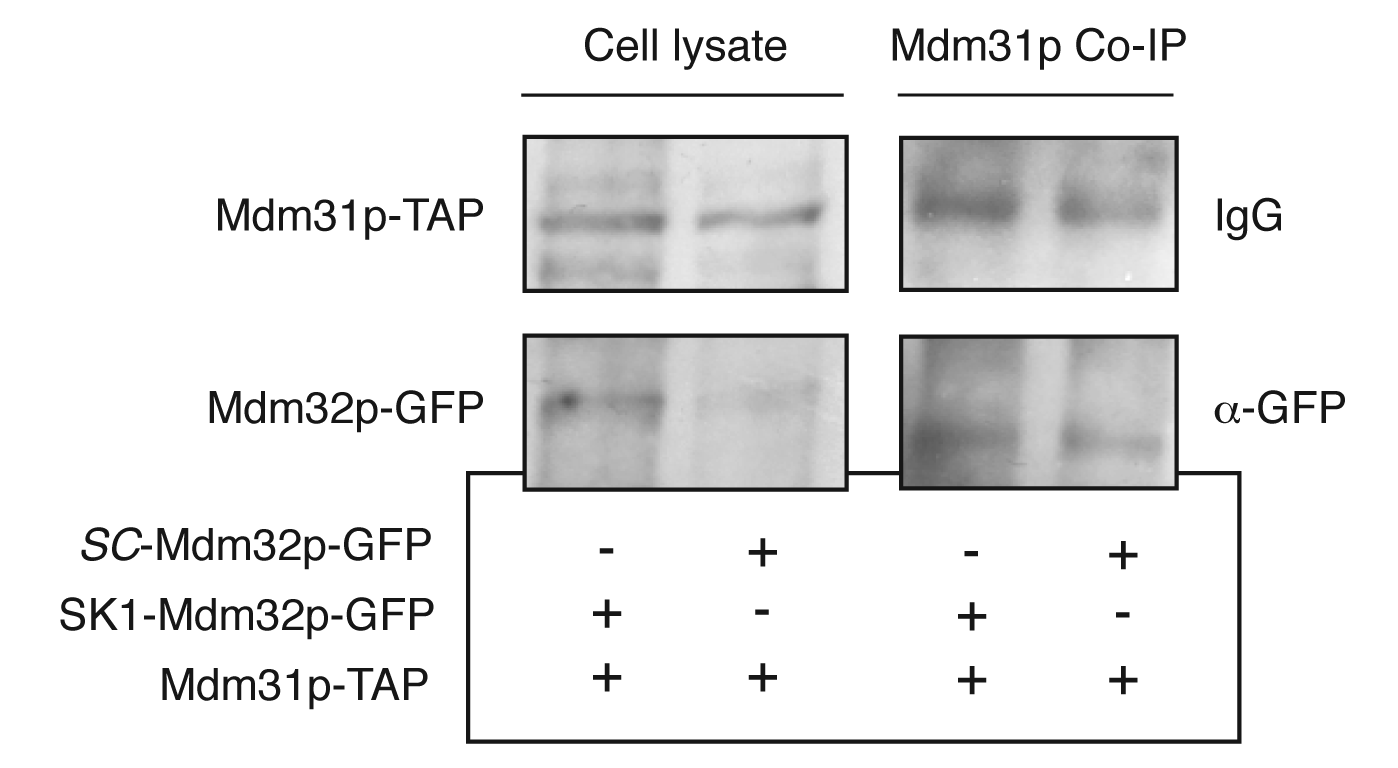

Supplement: Figure S3 — Binding of Mdm31p by BY4741- and SK1-encoded Mdm32p variants. The MDM32 open reading frames from SK1 and BY4741 were cloned into a GPD-eGFP Gateway plasmid, such that the MDM32 sequence was expressed from the GPD1 promoter as an in-frame 3′-fusion to sequence encoding enhanced GFP. MDM31 was cloned into the GPD-TAP plasmid, yielding a fusion of the tandem affinity purification (TAP) tag to the carboxy terminus of Mdm31p upon expression from the GPD1 promoter. The resulting plasmids were transformed into mdm32Δ mutants. The binding affinity between Mdm32p-GFP and Mdm31p-TAP was revealed by IgG pull-down using Mdm31p-TAP as bait. No significant difference in binding between Mdm31p-TAP and the respective Mdm32p-GFP variants was observed. (TIF) [file pgen.1004570.s003.tif]
