# Supplementary material for: Pooled Segregant Sequencing Reveals Genetic Determinants of Yeast Pseudohyphal Growth
Source: PLoS Genet. 2014 Aug 21;10(8):e1004570. doi: 10.1371/journal.pgen.1004570 (PMC4140661; doi:10.1371/journal.pgen.1004570)
Supplement: Table S1 — Genes exhibiting allelic linkage (LOD>3) from the BY4741-by-Σ1278b cross. Alleles in a single chromosome are demarcated by a double line; groupings of alleles within a possible linkage block are separated by a single line. (DOCX) [file pgen.1004570.s004.docx]

Table S1. Genes exhibiting allelic linkage (LOD >3) from the BY4741-by-Σ1278b cross

| Gene | Chr. | Nucleotide position | Allelic change | AA change | LOD |
| --- | --- | --- | --- | --- | --- |
| *UIP3* | I | 183875 | T-C | W-R | 3 |
| *ALT2* | IV | 678771 | A-G | D-G | 3.2 |
| *HPR1* | IV | 730732 | A-G | K-E | 3.6 |
| *YER046W-A* | V | 243718 | G-T | R-L | 4.1 |
| *PTP3* | V | 310095 | A-T | K-N | 3.2 |
| *YER076C* | V | 312722; 312806; 312830; 312962; 313202; 313340 | G-T; G-A; G-A; C-G; G-T; C-G | G-V; G-E; S-N; A-G; S-I; S-C | 4.6 |
| *YER076W-A* | V | 313483 | T-A | I-N | 3.9 |
| *YER077C* | V | 314585; 314943; 314975; 316218 | G-A; G-C; G-A; G-A | R-H; V-L; R-K; A-T | 3.6 |
| *ICP55* | V | 318305 | A-G | M-V | 3.1 |
| *AIM9* | V | 321040; 321397; 321775 | C-T; T-C; A-G | A-V; V-A; N-S | 3.6 |
| *YER084W* | V | 327359 | A-C | D-A | 3.4 |
| *YER084W-A* | V | 327966 | G-A | G-S | 5.1 |
| *DOT6* | V | 333269; 333440; 333615; 333665; 333684; 334212; 334228; 334313; 334739; 334746; 334877 | C-A; C-A; G-A; G-C; T-C; G-A; A-C; T-C; C-T; G-A; A-G | T-N; A-D; G-S; S-T; C-R; V-I; R-S; I-T; A-V; A-T; N-S | 5 |
| *FLO8* | V | 377186 | T-C | x-W | 17.4 |
| *AVT6* | V | 400477; 400832 | A-G; G-A | N-S; A-T | 14.5 |
| *YER119C-A* | V | 400886 | C-T | R-W | 14.3 |
| *YER121W* | V | 402396 | A-T | K-M | 15.1 |
| *GLO3* | V | 403553; 403774 | A-G; A-G | K-E; N-S | 15.3 |
| *YCK3* | V | 404920 | T-G | C-G | 15.4 |
| *DSE1* | V | 407953; 408140 | C-A; G-C | N-K; G-A | 14.7 |
| *LCP5* | V | 415066; 415306 | G-A; T-C | G-E; L-S | 9.9 |
| *SAK1* | V | 417316; 418486; 418864; 418870; 419264; 419532; 419564; 419627; 420275; 420428 | A-G; A-G; A-G; G-A; G-A; T-G; G-A; T-G; A-G; A-G | N-D; I-V; T-A; G-S; S-N; I-M; R-H; I-R; Q-R; D-G | 8.6 |
| *SCC4* | V | 464044; 464098; 464159; 464159 | C-T; G-A; C-T; G-T | T-I; C-Y; H-Y; G-E | 3.4 |
| *PEA2* | V | 466241 | C-A | L-M | 3.1 |
| *SQT1* | IX | 379273 | A-C | E-A | 3.3 |
| *FLO11* | IX | 389946; 390272; 390741; 390776; 391025; 391760; 393114; 393135; 393153; 393333; 393356; 393392; 393420; 393654; 393656 | A-G; T-C; T-C; C-T; T-C; T-C; C-T; A-G; G-T; A-G; G-A; G-A; G-C; A-G; C-T; T-C | I-V; L-P; S-P; T-I; I-T; V-A; H-Y; N-D; D-Y; N-D; G-D; G-D; W-S; K-E; L-F; L-P | 4.2 |
| *YIR020C* | IX | 394368 | G-C | V-L | 4.4 |
| *YIR020W-A* | IX | 394989; 395008; 395133 | A-G; T-C; A-G | T-A; V-A; T-A | 3.2 |
| *YIR021W-A* | IX | 398580 | G-C | A-P | 4.1 |
| *GAL80* | XIII | 171896 | A-C | E-D | 4.7 |
| *RSE1* | XIII | 176006 | G-A | R-K | 3.6 |
| *RCE1* | XIII | 814437; 815080 | G-A; A-C | V-I; L-F | 3.9 |
| *BUL1* | XIII | 815904; 818234; 818477 | G-A; G-A; T-C | G-S; G-E; V-A | 3.4 |
| *DSK2* | XIII | 819064 | C-G | A-G | 3.1 |
| *CAT8* | XIII | 827391; 829027 | T-C; A-G | L-S; T-A | 3.3 |
| *YMR290W-A* | XIII | 851619 | T-A | S-T | 3.2 |
| *MET4* | XIV | 428863 | T-C | I-T | 3.2 |
| *POL1* | XIV | 433150; 433708 | G-A; T-A | R-K; I-K | 3.1 |
| *YNL095C* | XIV | 446679 | C-G | S-C | 3.6 |
| *APP1* | XIV | 449116 | G-A | A-T | 4.2 |
| *YNL092W* | XIV | 450997 | C-A | A-E | 3.6 |
| *NST1* | XIV | 455104 | A-G | N-D | 3.1 |
| *RHO2* | XIV | 456838 | T-G | F-C | 3.3 |
| *YNL089C* | XIV | 456838 | A-C | K-Q | 3.3 |
| *MKT1* | XIV | 468490 | A-G | K-R | 3 |
| *NIS1* | XIV | 479818 | T-G | L-W | 3.3 |
| *CLN2* | XVI | 65676 | G-A | M-I | 3.2 |
| *ICY2* | XVI | 74459 | G-T | M-I | 3.1 |
| *FAS2* | XVI | 114050 | G-A | S-N | 3 |
| *TYW1* | XVI | 160842 | C-T | S-L | 3.6 |
