# Supplementary material for: Pooled Segregant Sequencing Reveals Genetic Determinants of Yeast Pseudohyphal Growth
Source: PLoS Genet. 2014 Aug 21;10(8):e1004570. doi: 10.1371/journal.pgen.1004570 (PMC4140661; doi:10.1371/journal.pgen.1004570)
Supplement: Table S2 — Genes assayed for deletion phenotypes with respect to invasive growth in Σ1278b. Deletion phenotypes were assessed using standard plate-washing assays. (DOCX) [file pgen.1004570.s005.docx]

Table S2. Genes assayed for deletion phenotypes with respect to invasive growth in Σ1278b

| Deletion mutant | Chromosome | Invasive growth phenotype |
| --- | --- | --- |
| *hpr1*Δ | I | Invasive |
| *dse1*Δ | V | Invasive |
| *sak1*Δ | V | Invasive |
| *pea2*Δ | V | Decreased invasive growth |
| *yer121w*Δ | V | Invasive |
| *yer046w-a*Δ | V | Invasive |
| *yir021w-a*Δ | IX | Decreased invasive growth |
| *cat8*Δ | XIII | Invasive |
| *dsk2*Δ | XIII | Invasive |
| *cyt1*Δ | XV | Increased invasive growth |
| *rho2*Δ | XIV | Decreased invasive growth |
| *ynl095c*Δ | XIV | Decreased invasive growth |
| *ynl092w*Δ | XIV | Invasive |
| *app1*Δ | XIV | Invasive |
| *nis1*Δ | XIV | Invasive |
| *icy2*Δ | XVI | Invasive |
