# Supplementary material for: Pooled Segregant Sequencing Reveals Genetic Determinants of Yeast Pseudohyphal Growth
Source: PLoS Genet. 2014 Aug 21;10(8):e1004570. doi: 10.1371/journal.pgen.1004570 (PMC4140661; doi:10.1371/journal.pgen.1004570)
Supplement: Table S3 — Genes exhibiting allelic linkage (LOD>4) from the BY4741-by-SK1 cross. Alleles grouped in a single chromosome are indicated by a double line; groupings of alleles within a single linkage block are indicated with a single line. (DOCX) [file pgen.1004570.s006.docx]

Table S3. Genes exhibiting allelic linkage (LOD >4) from the BY4741-by-SK1 cross

| Gene | Chr. | Nucleotide position | Allelic change | AA change | LOD |
| --- | --- | --- | --- | --- | --- |
| *SCM3* | IV | 211461 | C-T | T-I | 4.4 |
| *YDR524C-B* | IV | 1489710 | C-G | R-G | 4.3 |
| *AST2* | V | 361783 | A-C | E-A | 5.2 |
| *SSA4* | V | 366281 | G-A | R-K | 4.3 |
| *NUP157* | V | 368702; 369116; 370467; 370846; 371298; 371852; 371855; 371982 | T-C; G-A; G-A; G-A; A-G; A-C; G-A; A-G | F-S; G-E; A-T; M-I; N-D; K-T; R-K; I-V | 4.6 |
| *MAM1* | V | 372450; 373150 | G-C; A-C | K-M; T-P | 4.4 |
| *FLO8* | V | 375425; 376046; 376200 | G-A; T-C; A-G | S-N; I-T; T-A | 4.3 |
| *SLX8* | V | 385710; 395782; 396134 | A-C; G-A; G-A | K-N; M-I; G-D | 4.9 |
| *BOI2* | V | 392108; 393020; 393626 | G-A; C-T; C-T | S-N; P-L; S-L | 4.7 |
| *SPR6* | V | 394353 | T-C | C-R | 4 |
| *RPL23B* | V | 397397 | A-G | L-L | 7 |
| *SHO1* | V | 398971 | A-C | K-N | 4.7 |
| *AVT6* | V | 400447 | A-G | N-S | 4.1 |
| *YER119C-A* | V | 401102 | C-A | G-V | 5.5 |
| *SCS2* | V | 401855 | T-G | F-C | 6.1 |
| *YER121W* | V | 402396 | A-T | K-M | 7.7 |
| *GLO3* | V | 403533; 403774; 404059 | A-G; A-G; T-C | K-E; N-S; L-S | 5.1 |
| *YCK3* | V | 404920 | T-G | C-G | 6.1 |
| *DSE1* | V | 407415; 407953; 408140 | A-G; C-A; G-C | I-V; N-K; G-A | 5.3 |
| *LCP5* | V | 415066; 415306; 415480 | G-A; T-C; A-T | G-E; L-S; E-V | 4.8 |
| *SAK1* | V | 417316; 418509; 418870; 419224; 419532; 419564; 419627; 420275; 420592 | A-G; A-T; G-A; G-C; T-G; G-A; T-G; A-G; C-A | N-D; K-N; G-S; A-P; I-M; R-H; I-R; Q-R; H-N | 5.5 |
| *COM2* | V | 421715; 422222 | G-A; T-G | R-K; V-G | 4.5 |
| *RPS26B* | V | 424025 | T-C | F-L | 4.6 |
| *PMD1* | V | 425330; 426273 | T-C; A-T | S-P; Q-H | 5.6 |
| *YER133W-A* | V | 437262; 437412 | T-C; C-T | F-L; R-C | 4.3 |
| *YER134C* | V | 437412 | G-A | D-N | 4.3 |
| *YER135C* | V | 438034 | T-C | S-P | 4.2 |
| *YER137C* | V | 441621; 441759 | G-A; G-A | M-I; M-I | 5.3 |
| *YER137W-A* | V | 441828; 441839; 441866 | C-A; A-G; T-A | L-F; Y-C; I-K | 4.8 |
| *YER140W* | V | 452949 | T-C | F-L | 5 |
| *MAG1* | V | 455671 | A-G | I-V | 6.1 |
| *UBP5* | V | 458081; 458599; 459180; 459491; 459527; 459692; 459735; 459998 | C-T; T-A; T-C; C-T; A-G; G-A; G-A; G-C | S-F; D-E; S-P; T-I; D-G; R-K; A-T; G-A | 4.3 |
| *AAD6* | VI | 14848 | T-C | L-P | 4.2 |
| *EPS1* | IX | 347114 | A-G | I-V | 4.2 |
| *MPH1* | IX | 357797; 359491 | G-A; G-A | S-N;M-I | 4.1 |
| *AIM21* | IX | 361350 | G-A | E-K | 4.3 |
| *HAL5* | X | 108137 | T-C | V-A | 4.1 |
| *HSP150* | X | 120967 | G-A | V-I | 4.5 |
| *PRM1* | XIV | 111795 | T-A | H-Q | 4 |
| *CAF120* | XIV | 113719 | C-G | T-R | 4.2 |
| *YNL277W-A* | XIV | 116758 | G-A | G-E | 4.5 |
| *BOR1* | XIV | 119276; 119890 | T-A; T-A | N-K; I-N | 4.1 |
| *TOF1* | XIV | 124765; 125950 | G-A; G-A | G-D; G-E | 4.6 |
| *BNI1* | XIV | 130096; 132375 | A-G; G-A | I-M; E-K | 4.5 |
| *PIK1* | XIV | 142664; 142665 | G-A; G-A | G-S; G-D | 4.2 |
| *YIF1* | XIV | 147717 | G-C | G-A | 4.9 |
| *POL2* | XIV | 154540 | T-C | Y-H | 5 |
| *DSL1* | XIV | 159121; 159158; 159167 | G-C; C-T; T-A | E-D; A-V; L-H | 4.5 |
| *SIP3* | XIV | 160631; 161278; 162495 | G-C; T-C; G-T | A-P; M-T; L-F | 4.6 |
| *FOL1* | XIV | 165065; 165506; 165718; 165983; 166577; 166584 | G-A; A-G; A-T; T-A; A-G; C-T | V-I; N-D; E-D; L-I; R-G; A-V | 4.5 |
| *RTC4* | XIV | 168579 | T-C | V-A | 4.4 |
| *TEX1* | XIV | 170791 | A-G | E-G | 4 |
| *RAD50* | XIV | 176375; 177300; 178096; 178273 | A-G; A-G; G-A; A-G | D-G; I-M; D-N; I-V | 4.4 |
| *MPA43* | XIV | 180743 | G-T | R-L | 4.6 |
| *ATG2* | XIV | 191917 | T-C | I-T | 4.3 |
| *YNL057W* | XIV | 516537 | C-T | A-V | 4.5 |
| *YNL058C* | XIV | 516537 | G-A | A-T | 4.5 |
| *RIO1* | XV | 548996 | G-A | G-R | 4.9 |
| *GCY1* | XV | 551235 | A-G | T-A | 4.1 |
| *LEO1* | XV | 553519 | A-C | K-N | 4.1 |
| *UBP2* | XV | 556317; 558129; 558498 | A-G; A-T; C-T | D-G; D-V; T-I | 5.8 |
| *CAT5* | XV | 559460 | A-G | I-M | 5.2 |
| *RGA1* | XV | 562242; 562413; 563143; 563767 | C-A; A-T; A-C; T-C | P-T; I-F; Y-S; V-A | 5.5 |
| *ADE2* | XV | 565892 | A-G | R-G | 4.7 |
| *AFI1* | XV | 568463; 569111 | T-A; T-C | V-D; I-T | 5.9 |
| *ORT1* | XV | 570760 | A-G | I-V | 5 |
| *VPS17* | XV | 573249; 573359; 573378; 574379 | A-G; A-G; A-G; G-A | D-G; I-V; E-G; A-T | 5.5 |
| *BAG7* | XV | 578916 | G-A | D-N | 6 |
| *IRC14* | XV | 580334 | G-C | L-F | 7.1 |
| *SIA1* | XV | 581864 | C-T | L-F | 6.3 |
| *RUP1* | XV | 584519; 585809; 586077 | G-A; A-G; T-A | E-K; T-A; D-E | 7.3 |
| *YOR139C* | XV | 587197; 587207 | A-T; C-T | R-S; S-L | 8.8 |
| *SFL1* | XV | 587207; 588023; 588410; 588537; 589133 | G-A; G-A; C-T; T-C; A-G | E-L; V-M; Q-x; L-P; T-A | 7.8 |
| *ARP8* | XV | 590261; 592239; 592386 | T-A; G-A; G-A | D-E; G-E; G-D | 6.7 |
| *ELG1* | XV | 603167; 603550; 604120; 604414; 604516; 604573; 604763; 604805 | T-A; A-G; G-C; T-C; G-A; T-A; C-G; G-A | Y-N; E-G; S-T; V-A; R-H; F-Y; L-V; D-N | 5.7 |
| *MDM32* | XV | 607152; 607393 | G-C; A-T | C-S; L-F | 9 |
| *SPP2* | XV | 608819 | A-T | K-M | 5.6 |
| *SMP3* | XV | 609994; 610270; 610455; 610497 | A-G; G-A; A-G; C-A | M-V; D-N; Q-R; T-N | 4.9 |
| *MRPL23* | XV | 612124 | G-A | R-K | 5.7 |
| *PDR5* | XV | 619862 | A-G | N-D | 4 |
| *SLP1* | XV | 625879 | G-A | D-N | 4.7 |
| *YPL277C* | XVI | 15418 | G-C | R-P | 4.4 |
| *OPI11* | XVI | 654754 | G-A | G-S | 4.2 |
| *RPL43A* | XVI | 654754 | C-T | R-C | 4.2 |
| *YPR045C* | XVI | 656529 | C-A | H-Q | 4.5 |
| *ATG11* | XVI | 662621; 663705 | C-T; G-A | P-S; M-I | 4.5 |
| *YPR050C* | XVI | 665341 | A-G | K-R | 5.5 |
| *BRR1* | XVI | 672812 | G-C | E-D | 4.1 |
