# Supplementary material for: Pooled Segregant Sequencing Reveals Genetic Determinants of Yeast Pseudohyphal Growth
Source: PLoS Genet. 2014 Aug 21;10(8):e1004570. doi: 10.1371/journal.pgen.1004570 (PMC4140661; doi:10.1371/journal.pgen.1004570)
Supplement: Table S4 — Genes assayed for deletion phenotypes with respect to invasive growth in SK1. Deletion phenotypes were assessed for invasive growth by plate-washing assays. (DOCX) [file pgen.1004570.s007.docx]

Table S4. Genes assayed for deletion phenotypes with respect to invasive growth in SK1

| Deletion mutant | Chromosome | Invasive growth phenotype |
| --- | --- | --- |
| *sho1*Δ | V | Decreased invasive growth |
| *boi2*Δ | V | Invasive |
| *hal5*Δ | X | Decreased invasive growth |
| *mpa43*Δ | XIV | Decreased invasive growth |
| *gcy1*Δ | XV | Decreased invasive growth |
| *vps17*Δ | XV | Decreased invasive growth |
| *sfl1*Δ | XV | Invasive |
| *mdm32*Δ | XV | Decreased invasive growth |
| *mrpl23*Δ | XV | Decreased invasive growth |
| *idh2*Δ | XV | Decreased invasive growth |
| *brr1*Δ | XVI | Decreased invasive growth |
