# Supplementary material for: Pooled Segregant Sequencing Reveals Genetic Determinants of Yeast Pseudohyphal Growth
Source: PLoS Genet. 2014 Aug 21;10(8):e1004570. doi: 10.1371/journal.pgen.1004570 (PMC4140661; doi:10.1371/journal.pgen.1004570)
Supplement: Table S5 — Alleles with variation between BY4741 and Σ1278b where the BY4741-encoded allele exhibits linkage with the invasive phenotype. Alleles within a given chromosome are separated by a double line. (DOCX) [file pgen.1004570.s008.docx]

Table S5. BY4741 alleles from the Σ1278b cross exhibiting linkage with the invasive phenotype

| Gene | Chr. | Nucleotide position | Allelic change | AA change | LOD |
| --- | --- | --- | --- | --- | --- |
| *ALT2* | IV | 678771 | A-G | D-G | 3.2 |
| *HPR1* | IV | 730732 | A-G | K-E | 3.6 |
| *RCE1* | XIII | 814437; 815080 | G-A; A-C | V-I; L-F | 3.9 |
| *BUL1* | XIII | 815904; 818234; 818477 | G-A; G-A; T-C | G-S; G-E; V-A | 3.4 |
| *DSK2* | XIII | 819064 | C-G | A-G | 3.1 |
| *CAT8* | XIII | 827391; 829027 | T-C; A-G | L-S; T-A | 3.3 |
| *YMR290W-A* | XIII | 851619 | T-A | S-T | 3.2 |
| *CLN2* | XVI | 65676 | G-A | M-I | 3.2 |
| *ICY2* | XVI | 74459 | G-T | M-I | 3.1 |
| *FAS2* | XVI | 114050 | G-A | S-N | 3 |
| *TYW1* | XVI | 160842 | C-T | S-L | 3.6 |
