# Supplementary material for: Pooled Segregant Sequencing Reveals Genetic Determinants of Yeast Pseudohyphal Growth
Source: PLoS Genet. 2014 Aug 21;10(8):e1004570. doi: 10.1371/journal.pgen.1004570 (PMC4140661; doi:10.1371/journal.pgen.1004570)
Supplement: Table S6 — Alleles with variation between BY4741 and SK1 where the BY4741-encoded allele exhibits linkage with the invasive phenotype. Alleles within a given chromosome are separated by a double line. (DOCX) [file pgen.1004570.s009.docx]

Table S6. BY4741 alleles from the SK1 cross exhibiting linkage with the invasive phenotype

| Gene | Chr. | Nucleotide position | Allelic change | AA change | LOD |
| --- | --- | --- | --- | --- | --- |
| *SCM3* | IV | 211461 | C-T | T-I | 4.4 |
| *AAD6* | VI | 14848 | T-C | L-P | 4.2 |
| *HAL5* | X | 108137 | T-C | V-A | 4.1 |
| *HSP150* | X | 120967 | G-A | V-I | 4.5 |
| *PRM1* | XIV | 111795 | T-A | H-Q | 4 |
| *CAF120* | XIV | 113719 | C-G | T-R | 4.2 |
| *YNL277W-A* | XIV | 116758 | G-A | G-E | 4.5 |
| *BOR1* | XIV | 119276; 119890 | T-A; T-A | N-K; I-N | 4.1 |
| *TOF1* | XIV | 124765; 125950 | G-A; G-A | G-D; G-E | 4.6 |
| *BNI1* | XIV | 130096; 132375 | A-G; G-A | I-M; E-K | 4.5 |
| *PIK1* | XIV | 142664; 142665 | G-A; G-A | G-S; G-D | 4.2 |
| *YIF1* | XIV | 147717 | G-C | G-A | 4.9 |
| *POL2* | XIV | 154540 | T-C | Y-H | 5 |
| *DSL1* | XIV | 159121; 159158; 159167 | G-C; C-T; T-A | E-D; A-V; L-H | 4.5 |
| *MPA43* | XIV | 180743 | G-T | R-L | 4.6 |
| *SIP3* | XIV | 160631; 161278; 162495 | G-C; T-C; G-T | A-P; M-T; L-F | 4.6 |
| *FOL1* | XIV | 165065; 165506; 165718; 165983; 166577; 166584 | G-A; A-G; A-T; T-A; A-G; C-T | V-I; N-D; E-D; L-I; R-G; A-V | 4.5 |
| *RTC4* | XIV | 168579 | T-C | V-A | 4.4 |
| *TEX1* | XIV | 170791 | A-G | E-G | 4 |
| *RAD50* | XIV | 176375; 177300; 178096; 178273 | A-G; A-G; G-A; A-G | D-G; I-M; D-N; I-V | 4.4 |
| *ATG2* | XIV | 191917 | T-C | I-T | 4.3 |
| *YPL277C* | XVI | 15418 | G-C | R-P | 4.4 |
