# Supplementary material for: Pooled Segregant Sequencing Reveals Genetic Determinants of Yeast Pseudohyphal Growth
Source: PLoS Genet. 2014 Aug 21;10(8):e1004570. doi: 10.1371/journal.pgen.1004570 (PMC4140661; doi:10.1371/journal.pgen.1004570)
Supplement: Table S7 — Strains used in this study. (DOCX) [file pgen.1004570.s010.docx]

Table S7. Strains used in this study

| Strain | Genotype | Source |
| --- | --- | --- |
| HLY337 | *MATα ura3-52 trp1-1* | G. Fink (MIT, MA) |
| Y826 | *MATα ura3-52 leu2*Δ*0* | M. Snyder (Stanford, CA) |
| BY4741 | *MATa his3*Δ*1 leu2*Δ*0 met15*Δ*0 ura3*Δ*0* | M. Snyder (Stanford, CA) |
| SK1 | *MATa ura3-52* | T. Wilson (UM, MI) |
| SK1-Trp^-^ | *MATa ura3-52 trp1*Δ*0*::*URA3* | This study |
| *pea2*Δ | *MATα ura3-52 leu2*Δ*0 pea2*Δ::*URA3* | This study |
| *pea2-M409L* | *MATα ura3-52 leu2*Δ*0 pea2-M409L* | This study |
| *rho2Δ* | *MATα ura3-52 leu2*Δ*0 rho2*Δ::*URA3* | This study |
| *mdm32Δ* | *MATα ura3-52 mdm32*Δ *trp1*Δ*0*::*URA3* | This study |
| *mrpl23Δ* | *MATα ura3-52 mrpl23*Δ::*URA3* | This study |
| *mpa43Δ* | *MATα ura3-52 mpa43*Δ::*URA3* | This study |
| *idh2Δ* | *MATα ura3-52 idh2*Δ::*URA3* | This study |
| *brr1Δ* | *MATα ura3-52 brr1*Δ::*URA3* | This study |
| *sho1Δ* | *MATα ura3-52* *sho1*Δ::*URA3* | This study |
| *hal5Δ* | *MATα ura3-52 hal5*Δ::*URA3* | This study |
| *gcy1Δ* | *MATα ura3-52 gcy1*Δ::*URA3* | This study |
| *vps17Δ* | *MATα ura3-52 vps17*Δ::*URA3* | This study |
| *sfl1*Δ | *MATa ura3-52 sfl1*Δ::*URA3* | This study |
| *boi2*Δ | *MATa ura3-52 boi2*Δ::*URA3* | This study |
| *hpr1*Δ | *MATα ura3-52 leu2*Δ*0 hpr1*Δ::*URA3* | This study |
| *dse1*Δ | *MATα ura3-52 leu2*Δ*0 dse1*Δ::*URA3* | This study |
| *sak1*Δ | *MATα ura3-52 leu2*Δ*0 sak1*Δ::*URA3* | This study |
| *yer121w*Δ | *MATα ura3-52 leu2*Δ*0 yer121w*Δ::*URA3* | This study |
| *yer046w-a*Δ | *MATα ura3-52 leu2*Δ*0 yer046w-a*Δ::*URA3* | This study |
| *yir021w-a*Δ | *MATα ura3-52 leu2*Δ*0 yir021w-a*Δ::*URA3* | This study |
| *cat8*Δ | *MATα ura3-52 leu2*Δ*0 cat8*Δ::*URA3* | This study |
| *dsk2*Δ | *MATα ura3-52 leu2*Δ*0 dsk2*Δ::*URA3* | This study |
| *cyt1*Δ | *MATα ura3-52 leu2*Δ*0 cyt1*Δ::*URA3* | This study |
| *ynl095c-a*Δ | *MATα ura3-52 leu2*Δ*0 ynl095c-a*Δ::*URA3* | This study |
| *ynl092w*Δ | *MATα ura3-52 leu2*Δ*0 ynl092w*Δ::*URA3* | This study |
| *app1*Δ | *MATα ura3-52 leu2*Δ*0 app1*Δ::*URA3* | This study |
| *nis1*Δ | *MATα ura3-52 leu2*Δ*0 nis1*Δ::*URA3* | This study |
| *icy2*Δ | *MATα ura3-52 leu2*Δ*0 icy2*Δ::*URA3* | This study |
