# Supplementary material for: Pooled Segregant Sequencing Reveals Genetic Determinants of Yeast Pseudohyphal Growth
Source: PLoS Genet. 2014 Aug 21;10(8):e1004570. doi: 10.1371/journal.pgen.1004570 (PMC4140661; doi:10.1371/journal.pgen.1004570)
Supplement: Table S8 — Plasmids used in this study. (DOCX) [file pgen.1004570.s011.docx]

Table S8. Plasmids used in this study

| Plasmid | Description | Source or reference |
| --- | --- | --- |
| pLG669-Z-*FLO11*-6/7 | *P*_FRE_-_6/7_::*lacZ*, *URA3*, 2μm, Amp^r^ | Rupp *et al.* |
| pLG669-Z-*FLO11*-9/10 | *P*_FRE-9/10_::*lacZ*, *URA3*, 2μm, Amp^r^ | Rupp *et al.* |
| YCp-*PEA2*-Σ1278b | *P*_pea2_-*PEA2*(Σ1278b), *URA3*, Amp^r^ | This study |
| YCp-*PEA2*-S288C | *P*_pea2_-*PEA2*(S288c), *URA3*, Amp^r^ | This study |
| YCp-*MDM32*-SK1 | *P*_mdm32_-*MDM32*(SK1), *URA3,* Amp^r^ | This study |
| YCp-*MDM32*-S288C | *P*_mdm32_-*MDM32*(S288C), *URA3,* Amp^r^ | This study |
| pCu-HA-SPA2 | *P*_cup1_-*SPA2*, *URA3*, 2μ, Amp^r^ | This study |
| pCu-GFP-BUD6 | *P*_cup1_-*BUD6*, *HIS3*, 2μ, Amp^r^ | This study |
| YCp-GPD-*MDM31*-eGFP | *P*_gpd1_-*MDM31, URA3*, Amp^r^ | This study |
| YCp-GFP-*MDM32*-TAP | *P*_gpd1_-*MDM32, URA3*, Amp^r^ | This study |
